# A guided multiverse study of neuroimaging analyses

**DOI:** 10.1038/s41467-022-31347-8

**Published:** 2022-06-29

**Authors:** Jessica Dafflon, Pedro F. Da Costa, František Váša, Ricardo Pio Monti, Danilo Bzdok, Peter J. Hellyer, Federico Turkheimer, Jonathan Smallwood, Emily Jones, Robert Leech

**Affiliations:** 1 Centre for Neuroimaging Sciences, King’s College London, London, UK; 2 Center for Brain and Cognitive Development, Birkbeck College, London, UK; 3 Gatsby Computational Neuroscience, University College London, London, UK; 4 Department of Biomedical Engineering, McGill University, Montreal, Canada; 5 Mila - Quebec Artificial Intelligence Institute, Montreal, QC Canada; 6 Department of Psychology, Queen’s University, Kingston, ON Canada

**Keywords:** Neuroscience, Computational neuroscience, Cognitive neuroscience

## Abstract

For most neuroimaging questions the range of possible analytic choices makes it unclear how to evaluate conclusions from any single analytic method. One possible way to address this issue is to evaluate all possible analyses using a multiverse approach, however, this can be computationally challenging and sequential analyses on the same data can compromise predictive power. Here, we establish how active learning on a low-dimensional space capturing the inter-relationships between pipelines can efficiently approximate the full spectrum of analyses. This approach balances the benefits of a multiverse analysis without incurring the cost on computational and predictive power. We illustrate this approach with two functional MRI datasets (predicting brain age and autism diagnosis) demonstrating how a multiverse of analyses can be efficiently navigated and mapped out using active learning. Furthermore, our presented approach not only identifies the subset of analysis techniques that are best able to predict age or classify individuals with autism spectrum disorder and healthy controls, but it also allows the relationships between analyses to be quantified.

## Abstract (web summary)

Most neuroimaging studies are associated with a broad range analytic and methodological choices that the researcher needs to make, but every choice might lead to different answers, and evaluating all possible analytic choices is computationally challenging. Here, authors present a framework that maps the space of analysis by creating a low-dimensional space and using a Bayesian optimization to navigate it.

## Introduction

Typically, the research questions of neuroimaging studies are clearly specified (e.g., quantifying differences in functional connectivity measured with functional MRI (fMRI) between different groups or individuals), while the specific details of the analysis pipeline are not. For example, the analysis might vary in how the functional MRI data is processed to remove unwanted noise (e.g., which kernel, smoothing factor or type of motion correction to use), how the data is parcellated (e.g., anatomically, functionally defined reference brain atlases or data-driven approaches), or what specific analysis metric is selected to define connectivity (e.g., correlation, partial correlation) or quantify the data (e.g., which graph theory metric). Accordingly, it is now well recognized that a single research question can be addressed using a wide range of different analytic pipelines, often yielding slightly different answers^1–3^. In fledgling research areas, such as functional neuroimaging, in which many of the ground truths are yet to be discovered, analytic exploration is an unavoidable aspect of the scientific process. A central conceptual question facing the community, therefore, is how to balance the data exploration needed for scientific progress with the analytical rigor necessary to minimize the number of such discoveries that are false positives. To highlight this issue ref. ^4^ asked 70 independent teams to analyze the same dataset and test nine predefined hypotheses. Although all teams used different workflows to test these hypotheses and showed relatively high variability in the specific answers, a meta-analysis showed reasonable agreement among the broad results. The degree of consensus in studies such as this are important because different approaches can yield broadly similar answers and this, in turn, provides confidence that these conclusions are not tied to a specific analytic approach^5^.

Multiverse analyses are not only useful for finding consensus but for mapping interrelationships between pipelines more generally; in this article, we establish how mapping the inter-relationships between analyses can help understand homogeneity across different pipelines but also understand heterogeneity. Our study builds on prior studies that map the performance of different algorithms on different datasets^6^; in our study, we build a low dimensional representation of the analytic space of different pipelines. In the low-dimensional space, analytic pipelines that are closer produce similar outputs and therefore exhibit a more similar performance than pipelines that are further away in space. This structure helps build confidence in the conclusions drawn from specific analysis since it helps us understand how dependent specific results are on idiosyncratic aspects of the analytic approach. Exploring the space has other potential advantages; by combining pipelines (i.e., creating ensembles) that are further away in the multiverse space, the researcher can combine the strengths of different analysis pipelines. A better understanding of the space also allows a more informed characterization of the data as the researcher will be able to find areas where method developments may be needed.

Our analysis characterizes the multiverse as a low-dimensional space, which has important pragmatic benefits, in particular, it facilitates efficient search. There are near limitless potential analysis approaches and their combinations (i.e., pipelines), each with trade-offs between computational and time restrictions, as such fully mapping out the analysis space will not only impair the power of scientific inference but it can quickly become computational unfeasible. In a traditional approach, additional analyses reduce the sensitivity of statistical tests since it is important to correct for family wise errors that result from many comparisons. Mapping out the space in a low dimensional manner, while controlling the number of analyses sampled, has the potential for maintaining statistical power to detect effects. In our proposed multiverse approach, the trade-off between mapping the space efficiently and the number of analyses sampled is controlled by the researcher using the *κ* parameter (higher *κ* values result in a more detailed mapping of the space at the cost of computational and statistical power, lower *κ* values aim to find the best point in as few samples although will be more affected by local optima). The choice of this parameter will depend on the problem at hand and the desire to fully map the space or to obtain the best pipeline using only a few samples.

In summary, we present a framework that aims to map out the space of analysis pipelines efficiently, and that can maintain the sensitivity of inferential statistics and generalizability to out of sample data. Our approach allows us to explore many different features of the universe of pipelines and approaches, allowing many choices to be empirically compared without the need for exhaustive sampling. It does this through building a low-dimensional space across analysis pipelines, which is then mapped using Bayesian optimization^7^. This machine learning approach is flexible and the choice of how exploratory (mapping the space in detail) or exploitative (finding the best workflow with as few samples as possible) can be adapted depending on the research objective. We illustrate the utility of our multiverse approach in two contexts (1) a regression problem predicting age from functional connectivity obtained from adolescent and young adult participants^8^; and (2) a classification problem, predicting autism diagnosis from functional MRI. The motivation for using functional connectivity (FC) for this is two-fold: (i) FC applied to fMRI data is a useful technique for exploring the interrelationships between brain regions^9^; (ii) such approaches have also been shown to be highly sensitive to preprocessing steps such as motion correction, data parcellation, and analysis metric^10,11^. Moreover, there are dependencies between different types of measures (see ref. ^12^ for an example involving graph theory) such that the optimal analysis approach for any given dataset or question is typically unknown a priori. Focusing on the regression and classification problems allow us to evaluate the utility of the multiverse approach under a range of different conditions. We note, however, that the approach could be applied more generally to many different types of neuroimaging problems (both functional and structural) or indeed other types of data (e.g., univariate and multivariate analyses), and ultimately be applied to a range of basic scientific and clinical research.

## Results

### Regression analysis

**Fig. 1:**
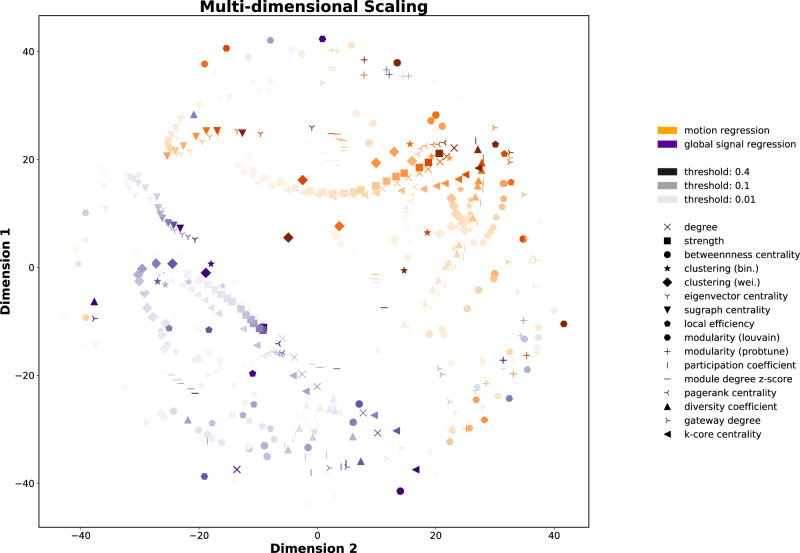
Regression analysis: low-dimensional embeddings of the different pipelines. Each point represents a combination of data accounting for noise confounds, thresholding of connectivity weights and different graph theory metrics. In particular, the colors represent both motion correction methods used to pre-process the data (i.e., motion regression (orange) and global signal regression (blue)), the color intensity represents the different thresholds used in each analysis and every graph theory metric is represented by a different symbol. The lower dimension space was obtained using Multi-dimensional scaling (MDS).

The first step is to construct a low-dimensional space from the analytic space. Figure 1 illustrates the space obtained using MDS. However, we also explored the obtained space using six additional embedding algorithms; the results are presented in the Supplementary Material (Fig. S1). All embedding algorithms demonstrate considerable structure in the position of the different approaches (e.g., similar types of motion correction, thresholding, graph metric are generally proximal). This suggests that the low-dimensional space captures the intended similarity between the approaches. We used a dissimilarity score to assess how much the different embedding algorithms preserved the topological information (i.e., similar pipelines should stay close to each other after the embedding; see the Supplementary Material). After varying number of k-Neighbors, multi-dimensional scaling (MDS) better maintained the neighborhood of the original space when *k* > 150. In addition, MDS displayed a relatively even spread of approaches across the whole space, especially when contrasted with Local-Linear-Embedding (LLE) and Spectral Entropy (SE). An approximately even spread across the space is desirable for the subsequent active learning and Gaussian process regression. As such, MDS was used in subsequent analyses.

**Fig. 2:**
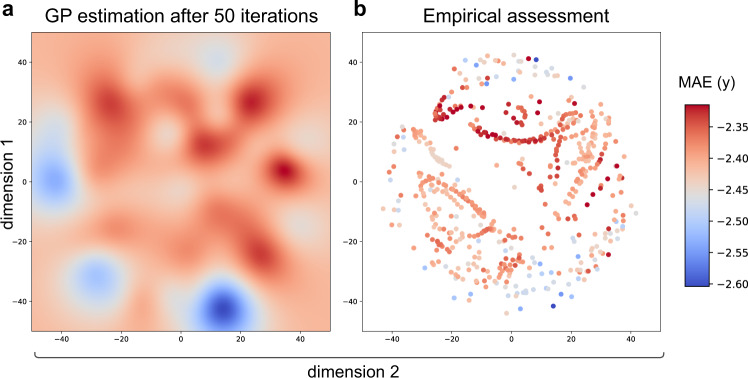
Regression analysis: learning the space and identification of the optimal analysis pipeline for age prediction. **a** After 50 iterations of Bayesian optimization, the Gaussian process (GP) using an exploratory sampling (*κ* = 10) closely estimates the empirical space. **b** Empirical assessment of age prediction across the whole space. The colors correspond to the negative mean absolute error (MAE) of each model in years. Values closer to zero represent a more accurate prediction and are shown in red.

**Fig. 3:**
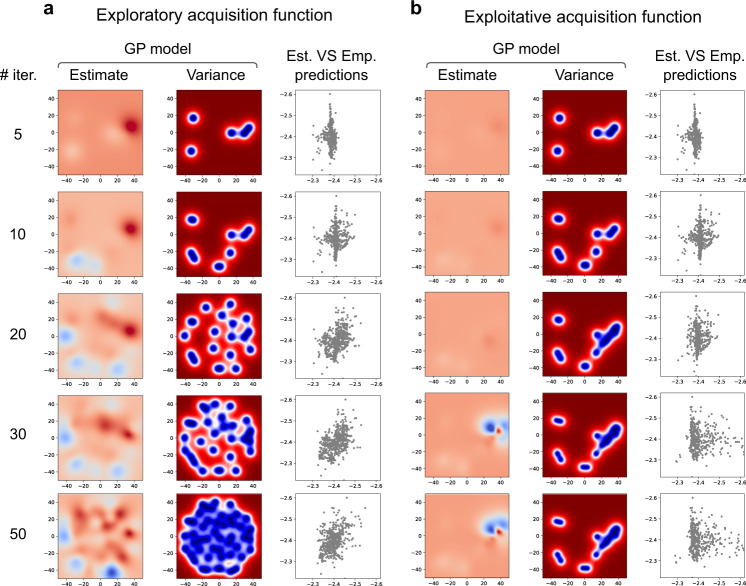
Regression analysis: the evolution of the search across the space for both the exploratory and exploitative function. **a** A more exploratory acquisition function (*κ* = 10); and, (**b**) a more exploitative acquisition function (*κ* = 0.1). Within each panel, the first column is the estimated Gaussian process (GP) model after different numbers of samples; the second column is the variance of the GP model across the space, indicating which points have been sampled; the third column is the estimated versus empirical predictions for all the pipelines in the space.

**Fig. 4:**
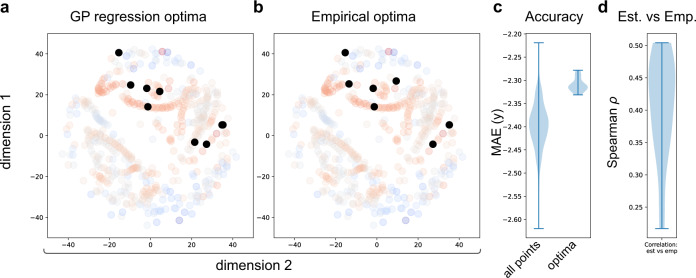
Regression analysis: performance of the optimization across different random starting conditions. For computational efficiency, only 20 iterations of active sampling were performed. Black dots represent optima of the 20 iterations based on (**a**) the highest accuracy estimated using the GP model and (**b**) the actual sampled points. **c** Range of negative mean absolute error for the optima versus negative mean absolute errors across the whole space. **d** Correlations between actual and estimated accuracies across the whole space for the 20 replications.

There are two objectives for the use of active learning on the MDS-defined space of different pipelines: (i) finding an approximately optimal analysis approach efficiently and controlling for the number of multiple comparisons; while, (ii) approximately estimating performance on the multiverse of approaches without exhaustive sampling. These two objectives can be observed in Figs. 2–4 where age-prediction models were trained and evaluated for different pipelines selected by active learning. As visible from Fig. 2, the range of predictions for all pipelines ranged between (MAE (years) [−2.62, −2.22]; (mean ± std = −2.39 ± 0.050)).

Figure 2 describes the result of the Gaussian process regression after 50 iterations of active learning. Based on the 50 different analysis approaches sampled, GP regression estimates performance across all 544 approaches (Fig. 2a); this identifies areas predicted to have higher age-prediction performance (in warm colors) including the optimum, as well as approaches which perform worse (in cooler colors). For comparison, the ground truth of performance across the space (from exhaustive sampling every approach) is presented in Fig. 2b. We observe a generally good concordance between actual age prediction for each approach and the estimated prediction across the whole space (Spearman’s *ρ* = 0.397, *p* < 0.0001).

The evolution of the active sampling and Gaussian process regression model is presented in Fig. 3. We initially observe a poor GP estimation of the space based on the first 10 random burn-in samples. As the sampling increases, the space is progressively better estimated achieving increasingly higher correlations between empirical and estimated spaces. Acquisition function parameters strongly affect the active sampling; to illustrate this, the parameter *κ* was varied to conduct both exploratory (*κ* = 10, Fig. 3a) and exploitative versions of active sampling (*κ* = 0.1, Fig. 3b). The exploratory version achieves a better estimation of the whole space, while the exploitative version focuses on an estimated optimum much more quickly, but the GP model changes much less subsequently, resulting in a much lower correlation between estimated and empirical accuracies across the space.

To investigate the reliability of the active sampling, the process was repeated 20 times (using the more exploratory *κ* = 10) with different random seeds (and so different initial random burn-in sampling). In Fig. 4, the optima (i.e., model with the highest empirical accuracy) of the 20 repetitions are represented by the black dots, based both on the highest accuracy estimated using the GP model (Fig. 4a) and for the actual sampled points (Fig. 4b). Table 1 presents the optimal analysis approaches selected by each iteration. We note that many of the optima illustrated in Table 1 were obtained by using the Betweenness centrality. The range of the mean absolute error for the different optima selected versus the full range of mean absolute errors across the whole space is presented in Fig. 4c and the range of correlations between actual and estimated accuracies across the whole space for the 20 replications is presented in Fig. 4d.

**Table 1:** Regression Analysis: List of the data, threshold, graph theory metric and obtained mean absolute error (MAE) for the empirical optima obtained for the 20 iterations.

Data Regression	Sparsities	Graph Theory Metric	Mae
Motion Regression	0.125	gateway degree	−2.434
Motion Regression	0.060	betweennness centrality	−2.531
Motion Regression	0.250	eigenvector centrality	−2.442
Motion Regression	0.300	modularity (louvain)	−2.591
Motion Regression	0.050	strength	−2.419
Motion Regression	0.125	gateway degree	−2.408
Motion Regression	0.125	gateway degree	−2.408
Motion Regression	0.100	betweennness centrality	−2.388
Motion Regression	0.300	modularity (louvain)	−2.591
Motion Regression	0.100	betweennness centrality	−2.388
Motion Regression	0.100	betweennness centrality	−2.388
Motion Regression	0.100	betweennness centrality	−2.388
Motion Regression	0.100	betweennness centrality	−2.442
Motion Regression	0.100	betweennness centrality	−2.388
Motion Regression	0.100	betweennness centrality	−2.388
Motion Regression	0.100	betweennness centrality	−2.388
Motion Regression	0.100	betweennness centrality	−2.388
Motion Regression	0.020	module degree z-score	−2.528
Motion Regression	0.020	module degree z-score	−2.528
Motion Regression	0.020	module degree z-score	−2.528

### Classification analysis

**Fig. 5:**
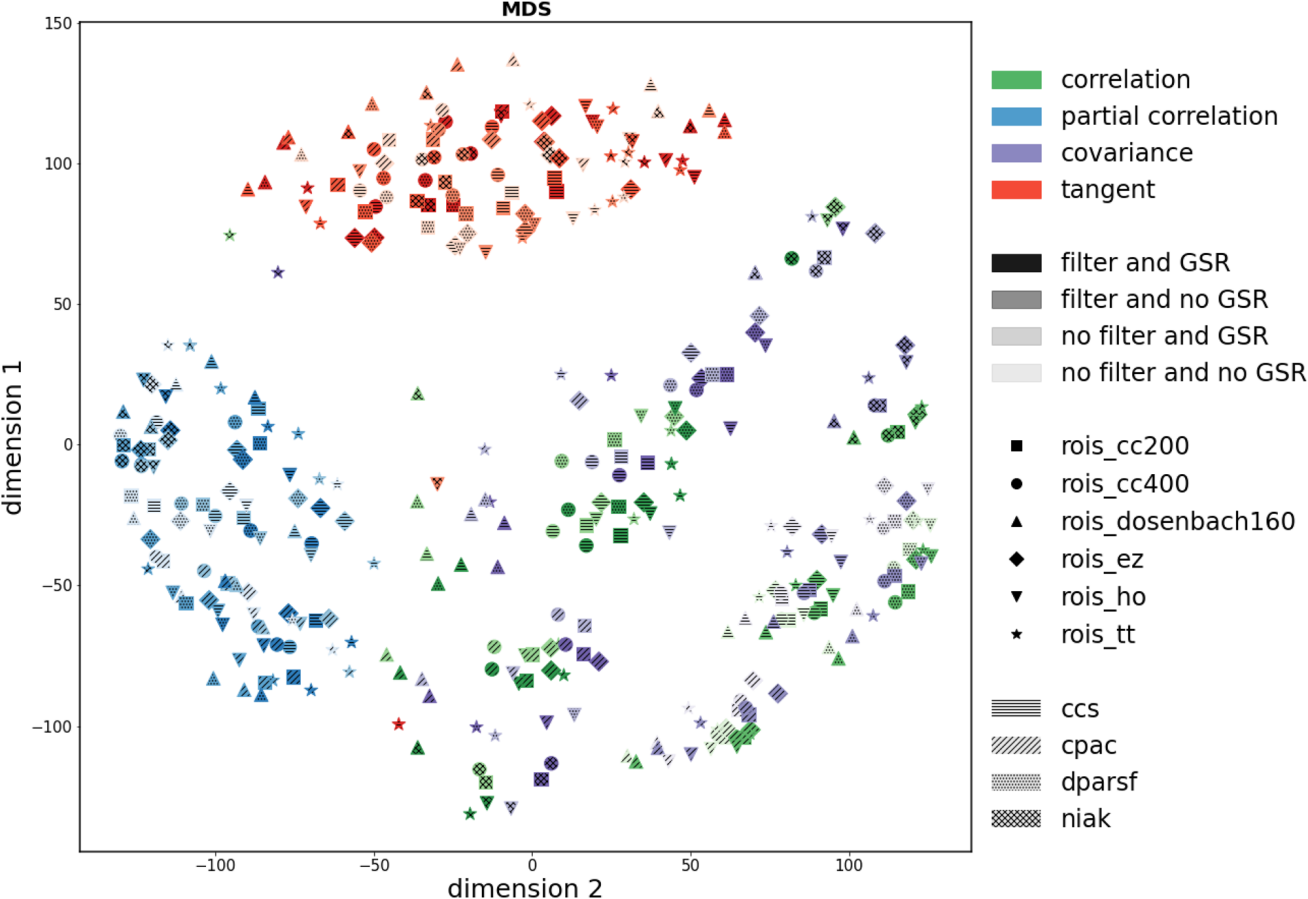
Classification analysis: low-dimensional embeddings of the different pipelines. Each point represents a combination of data accounting for four different function preprocessing steps (represented by the different hatch styles), processing strategy (the color intensity), ROIs (shapes) and connectivity metrics (different colors). The image illustrates the space generated using MDS. Despite having no knowledge about the space, the embedding algorithm was able to identify the structure of the space.

We also explored how the multiverse approach could be used when distinguishing between autistic people and controls. Similar to the process used on the regression analysis, the first step consisted of building a low-dimensional space that contained the information. While the results for the MDS approach are depicted in Fig. 5, the embedded space obtained by using LLE, SE, t-SNE, and UMAP are shown in Fig. S3 on the Supplementary Materials. Although all approaches were able to identify structure within the space, we choose MDS as it created an even spread across the space that is essential for active learning and used this method in further analyses. Figure 5 illustrates how despite being agnostic of the different pipelines, the approach identified the structure on the dataset and grouped similar pipelines together (e.g., the same connectivity metric tends to be clustered together).

**Fig. 6:**
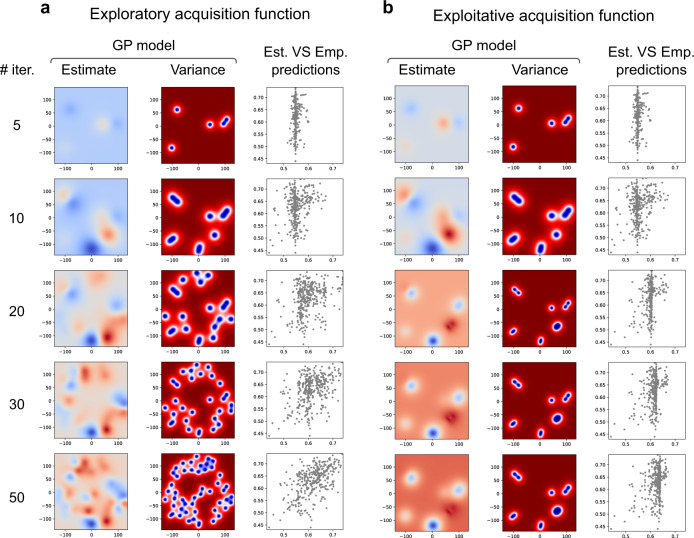
Classification analysis: the evolution of the search across the space for. **a** A more exploratory acquisition function (*κ* = 10); and, (**b**) a more exploitative acquisition (*κ* = 0.1) function. Within each panel, the first column is the estimated Gaussian Process (GP) model after different numbers of samples; the second column is the variance of the GP model across the space, indicating which points have been sampled; the third column is the estimated versus empirical predictions for all the pipelines in the space. After 50 iterations the exploratory analysis shows a good correlation (*ρ* = 0.55) with the empirical space.

The evolution of the search space is depicted in Fig. 6. Similar to the regression analysis the choice of the acquisition function (i.e., exploitative or exploratory sampling) greatly impacts the obtained results. The exploratory sampling (Fig. 6a) achieves a better estimation of the space compared to the exploitative acquisition function (Fig. 6b). Stating the relevance of pre-processing for prediction, the accuracy of the predictions on the classification space (measured by the AUC) varied between [0.4, 0.7] (0.65 ± 0.06 − mean ± std).

**Fig. 7:**
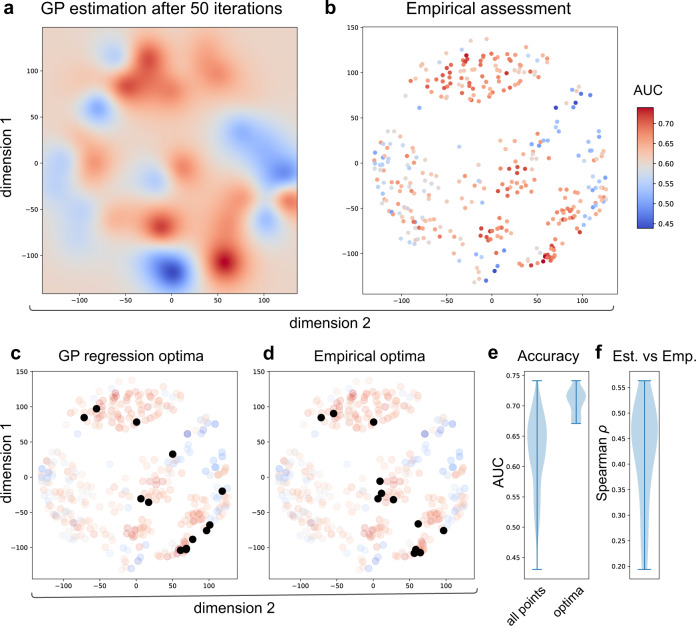
Classification analysis: the space of optimal pipelines for classifying healthy controls and autistic subjects using FC. The estimated space using 50 iterations of Bayesian optimization using an exploratory sampling (*κ* = 10) (**a**) and the empirical space obtained after assessing the entire space (**b**). The colors represent the performance of the classifier measured by the area under the curve (AUC). Values closer to 1 represent a more accurate prediction and are shown in red, values close to 0.5 indicates chance performance and are depicted in blue. To make sure that the optima obtained by the Bayesian optimization were not given by chance, we repeated the same analysis 20 times. The black dots depict the found optima by GP (**c**) and the actual empirical optima, which are only available by extensive sampling the space (**d**). The GP algorithm was able to identify the optimal zones by only evaluating a smaller range of pipelines. **e** Summary of the range of the performance obtained on the empirical assessment for all points and the accuracy of the optima points. **f** Spearman correlation between the empirical observed accuracy and the estimated optima for the 20 replications.

We observe a good overlap between the empirical space and the estimated space when using an exploratory approach (*κ* = 10) (Figs. 7a, b). Similar to the regression analysis, we also investigated the reliability of the activity sampling on the classification problem (Figs. 7c, d). The range of the AUC for optima found by the active learning is depicted in Fig. 7e and the correlation between the actual and estimated in Fig. 7f.

## Discussion

In this paper, we established that active sampling can be used to map out a low-dimensional space of the multiverse of analytic approaches allowing the processing pipelines with higher accuracy to be identified in an efficient manner. To understand the flexibility of our approach we focused on two questions that are often examined using resting state functional connectivity: a regression problem (predicting brain age) and a classification problem (distinguishing between individuals with autism spectrum disorder and controls). Since efficient exploratory research is critical for neuroimaging in order for it to become a mature scientific discipline, our multiverse approach is helpful because it balances the need for rapid discovery with an analytic approach that maps out the inter-relationships between the analyses in a low-dimensional space. Our approach, therefore, establishes multiverse approaches a cost effective method for balancing Type I and II errors in a range of neuroimaging contexts.

Our analysis applied active sampling to predict age and classify autism diagnosis from functional connectivity and by doing so illustrated why the multiverse approach is helpful. Our main aim was to showcase active sampling on a space of pipelines, rather than identify (the) optimal combination(s) of processing steps in general. Nevertheless, it is interesting to consider the general themes that emerge from the family of approaches selected as optimal by this approach. By repeating the active learning method 20 times, we see substantial consistency in processing steps across the selected optima for both the regression and classification situations. For the regression problem, motion regression consistently outperforms the global signal regression; lower, but not the lowest sparsities were also favored using a range of both simple and complex graph theoretical metrics, with betweenness centrality selected most frequently. These results are important because global signal regression is one of the most debated fMRI processing steps, with many arguments proposed both for and against its inclusion in processing pipelines^13,14^. With respect to thresholding we observed that most of the optima had a higher threshold. This is in line with previous research that observed that connections with lower edge weights (i.e., correlation) are more likely to be spurious suggesting that connectomes thresholded to lower densities might be less affected by noise^11,15^. Finally, as many of the optima illustrated in Table 1 were obtained by using the Betweenness centrality, this might suggest that this graph theory metric is more robust to the usage of different pre-processing choices. Betweenness centrality had previously been found to perform well in network neuroimaging applications, including in machine learning applications^16^. For the classification dataset, both in the organization of the low-dimensional space (Fig. 5) and the estimated optima (Fig. 7a), we observe that the method used to infer connectivity was the most salient organizing feature, with the tangent projection showing a more robust classification performance and independent of the other pre-processing steps using the tangent projection as the metric yielded better accuracy (consistent with what has previously been observed^17–19^). Other factors such as how the data was parcellated were more variable in terms of performance^20^. However, by repeating the active learning method 20 times we noticed that only a few repetitions (3/20—Table 2) were built using the tangent metric. This illustrates that there is not a single component of the pipeline that is likely to be superior across different goals, but that different questions may entail different combinations of the analytic components that might influence the pipeline’s performance in a specific context.

**Table 2:** Classification analysis: list of the functional pre-processing, ROIs, processing strategy, connectivity metric and the obtained score for the empirical optima obtained for the 20 repetitions.

Functional pre-processing	ROIS	Processing strategy	Connectivity metrics	Score
cpac	rois_cc200	nofilt_noglobal	correlation	0.720
ccs	rois_cc400	filt_global	correlation	0.740
ccs	rois_cc400	filt_global	correlation	0.740
dparsf	rois_ho	nofilt_global	tangent	0.696
dparsf	rois_ez	filt_noglobal	covariance	0.471
cpac	rois_ho	nofilt_global	tangent	0.637
ccs	rois_cc400	nofilt_global	correlation	0.756
cpac	rois_ez	filt_noglobal	correlation	0.670
cpac	rois_ho	filt_noglobal	tangent	0.645
ccs	rois_ez	nofilt_global	covariance	0.675
cpac	rois_tt	filt_noglobal	correlation	0.687
dparsf	rois_dosenbach160	filt_noglobal	correlation	0.605
dparsf	rois_dosenbach160	filt_noglobal	covariance	0.474
cpac	rois_ez	filt_noglobal	covariance	0.639
cpac	rois_ez	filt_noglobal	covariance	0.639
cpac	rois_ez	filt_noglobal	covariance	0.639
cpac	rois_ez	filt_noglobal	covariance	0.639
cpac	rois_ez	filt_noglobal	covariance	0.639
cpac	rois_ez	filt_noglobal	covariance	0.639
cpac	rois_ez	filt_noglobal	covariance	0.639

Abbreviations: ccs (Connectome Computation System); cpac (Configurable Pipeline for the Analysis of Connectomes); dparsf (Data Processing Assistant for Resting-State fMRI); filt (Band-pass filtering (0.01–01 Hz); global and noglobal (Global signal regression and no global signal regression, respectively)); rois_cc200 (ROI extraction using the Craddock 200 parcellation); rois_cc400 (ROI extraction using the Craddock 400 parcellation); rois_ho (ROI extraction using the Harvard-Oxford atlas); rois_ez (ROI extraction using the Eickhoff-Zilles atlas); rois_tt (ROI extraction using the Talaraich and Tournoux atlas); rois_dosenbach160 (ROI extraction using the Dosenbach 160 atlas);

Fortunately, our multiverse approach is highly flexible and so can be easily tailored to many different analytic goals. If a researcher is interested in applying our method to a new dataset, there are only two building blocks that need to be adapted: (1) It is necessary to create a low-dimensional space that quantifies the similarity of the different samples under analysis. We used the cosine similarity to quantify the difference among FC, however, different metrics could be used. The low-dimensional space does not require that the analyses result in data of the same format; it is possible to combine univariate and multivariate analyses (e.g., networks, single regions, or every voxel or vertex measured), and also different modalities. These features allow our multiverse approach to cover a very heterogeneous collection of approaches. (2) An active learning approach is needed to navigate the low-dimensional space. The active sampling component requires every analysis to be evaluated by a common set of target measures. In a predictive context, a cost function (e.g., classification accuracy, mean absolute error) can be used to evaluate pipelines; however, our proposed approach can also be applied to non-predictive measures, such as parameter estimates or standardized measures (e.g., effect size or t-statistics). Active sampling may also be applicable to a subset of non-supervised problems (e.g., without data labels/ group assignments). For example, a measure of test-retest reliability (e.g., the interclass correlation coefficient (ICC)) could be used as the target metric to navigate the space. That is instead of using the mean absolute error or the classification accuracy, as done in this paper, the researcher could use the ICC as a cost function. However, this choice would require a test and re-test acquisition of the dataset being used for the active sampling. In addition, our proposed approach could also be integrated with automated pipelines such as fMRIPrep^21^, to allow controlled, efficient exploration of a much wider range of preprocessing and analysis. As such, the approach is potentially relevant for many questions in neuroimaging. However, if a single target metric can not be defined (e.g., in descriptive or exploratory data analysis) or when there are multiple, target metrics that cannot be aggregated a priori into a single target metric then the approach proposed in this paper will not be suitable.

In the current paper, the analysis space was developed from a subset of the whole participant group; however, this need not be the case in all versions of this approach. A predefined space can be constructed using an existing dataset, and subsequently applied, with minimal computational cost, to different datasets. For example, large open datasets such as the Human Connectome Project^22^ or UK Biobank^23^ could be used to define analysis spaces which can then be applied to smaller (e.g., clinical) datasets. This would mirror the strategy taken with many deep learning approaches, which are computationally expensive to train but not to apply to new data. The success of such a strategy would depend on how much similarity there is across datasets and how these intersect with the specific analysis approaches taken. We note, however, that for active learning to be efficient it does not require a perfectly organized space, but rather the much weaker assumption that nearby points in the space are similar in terms of the target function (e.g., classification accuracy). As such, even a relatively weak mapping from a space defined on a larger dataset, may be sufficient for efficient sampling.

Furthermore, because the data was extensively cleaned before it was used in the pre-processing pipelines, we did not add any quality control checks to test if the pipelines lead to erroneous connectivity matrices for specific subjects. However, quality controls that either drop-out subjects or censure problematic time points, will be an interesting avenue for future extensions of our work. We note that a benefit of our approach is that it will not converge on pipelines that fail for many subjects as those will have a poor performance and will therefore not lie in the optimal areas in the space. Therefore, the low-dimensional space could be used to check the validity of a specific analysis step within a pipeline; if all pipelines using a specific analysis produced erroneous connectivity matrices in all or a large proportion of participants they will show high similarity and will be clustered closer together away from the optima.

Performing multiverse analyses has the potential for increasing the generalizability of results (e.g., ref. ^24^). As recently revisited by Yarkoni^25^, when interpreting findings, we often go (both statistically and verbally) far beyond what is justified by the restricted nature of the data and quantitative analyses performed. Our proposed approach helps overcome this problem by building more generalizable results in two ways: (1) assessing the generalizability of a pipeline or specific step of a pipeline compared to other pipelines. That is, how specific or general is the choice of a particular parameter in influencing performance. A good example would be the usage of the tangent connectivity matrix on the classification analysis, where the tangent projection both clearly clusters together (Fig. 5), and is associated with high performance (Fig. 7a). (2) Assessing the generalizability of pipelines to out of sample data and over different repetitions (Fig. 4 and Figs. 7c–f). By efficiently sampling the space, there is less opportunity for overfitting the data than exhaustively sampling all pipelines, maintaining generalizability to out of sample data. In addition, mapping out the space in this manner has other potential benefits; it allows us to know which areas of the space are undersampled (as a guide to developing new approaches) and which are oversampled or irrelevant to the problem. Furthermore, as the low-dimensional space captures the variability in connectivity maps, it is possible to identify more than one region in the low-dimensional space with good performance. The methods in these distinct areas are likely to capture different information on the data and could be further combined into ensembles.

The efficiency of the multiverse approach rests in the way that the same data is only used a limited number of times, reducing the problems inherent in sequential analyses in terms of both overfitting and false positives. In the extreme, it is possible to perform each iteration of the active sampling on a different subset of participants who are then not reused; as such, each suggestion from the Bayesian optimization for the next point to be sampled would involve out-of-sample prediction. How exploratory or exploitative the acquisition function is, will affect the number of samples and so the degree of possible over-fitting (and loss of statistical power). Fortunately, as our paper demonstrates, the choice of acquisition function can be motivated by the scientific question and the degree to which optimization of prediction or mapping out the analysis space is the objective.

Similarly to previous work using Bayesian optimization for the navigation of predefined experimental spaces^26–28^, the method presented here can help improve the poor reproducibility present across much of (neuro)science. Sequential analysis as applied here is highly formalized, quantifiable and controllable, and as such, it can be readily combined with pre-registration^29^. Furthermore, the route and samples taken by the analysis make it possible to deduce what the hypothesis (encoded as the target function of the optimization algorithm) was at the time of testing. If a different target function was selected, then the algorithm would have taken a different route through the analysis space (see ref. ^29^). This means that questionable research practices such as SHARKing may be more difficult to pursue^30^.

As with any analysis approach, using active sampling methodologies comes with inherent trade-offs. Most notably, for more exploitative problems, where the optimal analysis approach is known (or approximately known) a priori or highly theoretically constrained, then the additional costs (in terms of sequential analysis affecting statistical power and computational burden) are a serious limitation. The optimization algorithm finding local minima resulting in poor overall performance is another potential limitation; this will depend heavily on the acquisition function including the type used and hyperparameters controlling exploration and exploitation as well as decisions regarding the GP regression and types of kernels used to model the low-dimensional space. A related issue is the creation of the low-dimensional space itself; this will inevitably involve a trade-off between capturing relevant variance and creating a relatively simple search space, with few dimensions. We show that the search space is coherent (in terms of the placement of similar pipelines near each other—Fig. 1) and the GP regression is able to capture regularities in the space efficiently (Fig. 2). However, for other problems, e.g., involving lower signal-to-noise ratios, more heterogeneous variability across individuals, or more heterogeneous analysis approaches, building a compact search space may be more challenging. Future work is needed to find the most useful acquisition function, GP regression and search spaces for applying active sampling approaches to multiverse analyses.

In summary, we have presented a method for efficiently exploring outcomes across multiple analyses by building a low-dimensional space that captures the similarity of pipelines and subsequently exploring it using active learning. This maintains the sensitivity of inferential statistics and generalizability to out of sample data while mapping out the multiverse of pipelines, and so enhancing the efficiency of scientific discovery. Although we have illustrated this analysis and its efficiency using FC data on both a regression and classification problem, this approach could be applied to different neuroimaging problems (both functional and structural) or indeed other types of data.

## Methods

While the proposed framework can be applied to many neuroimaging studies, we focus on its capabilities in both a regression context to predict age, and in a classification context, to distinguish between controls and autistic participants. We chose these two problems to illustrate how the method could be applied in different settings (i.e., two supervised problem scenarios), however, the multiverse approach could be used to map the space of other problems and datasets depending on the researcher’s interest. The framework consists of two main steps: (i) create a low-dimensional continuous space of the different pipelines; (ii) an active learning component that efficiently searches the created space to find the optimal analysis pipeline and produces estimates of the performance of other pipelines.

All code used for analyses and figure generation is available on GitHub (https://github.com/Mind-the-Pineapple/into-the-multiverse). In the next sections, we describe the data, the range of approaches considered, how the analysis space was constructed and finally, the active learning approach used to sample the space and so be able to estimate brain age and classify controls and individuals with autism spectrum disorder across the multiverse of pipelines without exhaustive testing. Note that both datasets are available online and the ethics committee of the leading institution of each study approved it.

### Regression analysis

We first focused on predicting brain age as it has been proposed as a useful biomarker of neurological and psychiatric health^31,32^ and is predictive of a range of other factors, including mortality^33^. More generally, predicting age is a useful proof of principle for methodological demonstrations since the participant’s age is known with certainty^34^ and a range of studies have shown that functional connectivity from resting state correlates with age (e.g., refs. ^8,35,36^).

#### Functional connectivity data

The starting point for our regression analysis is a functional MRI dataset of changes in functional connectivity across adolescence from^8^. This dataset consists of 520 scans from 298 neurologically healthy individuals (age 14–26, mean age = 19.24, see^8^ for details). Here, we only performed cross-sectional analyses and so only kept the first scan for each individual. The dataset was split into three parts: (i) 50 individuals, selected at random, were used to build the low-dimensional space; (ii) 198 individuals were subsequently used to perform search and (iii) 50 individuals were used as a holdout dataset (or a “lock box approach”^37^) to test the best pipeline selected by active learning on an independent subset of the dataset.

#### High dimensional space of pipelines

There are many decisions necessary to conduct a functional connectivity study, including choices regarding data acquisition, pre-processing, summary metrics and statistical models. Here, for convenience, we use already acquired data which has been pre-processed using extensive pipelines to reduce many potentially confounding sources of non-neural artefacts. Usefully, two preprocessed datasets were shared by ref. ^8^ with two different types of correction for movement artefacts: (i) global signal regression and (ii) motion regression. The preprocessed fMRI time series had been averaged within 346 regions of interest, including 330 cortical regions from the Human Connectome Project multi-modal parcellation^38^ (excluding 30 “dropout” regions with low signal intensity) and 16 subcortical regions from Freesurfer. The Pearson correlation coefficient was used to calculate functional connectivity (FC) between these regions. For further details regarding data pre-processing, see ref. ^8^.

In order to demonstrate the capabilities of the proposed method we consider three distinct analysis pipeline choices. These are:

1. The nature of regression: we explored data from two types of data regression—motion regression and global signal regression.
2. The choice of threshold for estimated functional connectivity matrices: we consider 17 distinct threshold values ranging from 0.01 (resulting in highly sparse networks) to 0.4 (resulting in dense networks).
3. The graph theoretical metric studied: we consider 16 distinct simple and higher-level features of network organization (see the Supplementary Material (Table S1) for a full list) that have been successfully employed in previous neuroimaging studies. Metrics were calculated using the Python implementation of the Brain Connectivity Toolbox^39^; only nodal metrics were included. If prior community assignment information was required, we used the well-known Yeo network parcellation of the brain into seven networks^40^.

The full space of analysis options is presented in Table S1. Every single evaluated pipeline was built by using one of the regression choices, one threshold and one graph theory metric. Therefore, the analyzed multiverse space consisted of 2 × 16 × 17 = 544 different pipelines.

### Classification analysis

To further test the effectiveness of our proposed method, we evaluated how well our estimated space could be used to distinguish between controls and individuals with autism spectrum disorder using the ABIDE dataset^41^. The already preprocessed dataset using different pipelines and parameters was downloaded from the Preprocessed Connectome Project (http://preprocessed-connectomes-project.org/abide). A detailed description of how the data was processed can be found at (http://preprocessed-connectomes-project.org/abide/Pipelines.html).

#### Functional connectivity data

The used dataset contained functional connectivity data from 882 subjects (476 neurotypical controls and 406 autistic people; mean age = 17.19). Similar to the regression analysis the dataset was split into: (i) 176 subjects to build the low-dimensional space; (ii) 529 subjects to search the created space, and (iii) 177 individuals were held back as a lock box^37^. We used a stratified split to ensure that the distribution between controls and individuals with autism spectrum disorder was maintained throughout the splits.

#### High dimensional space of pipelines

Similar to the regression analysis described above, we analyzed different pre-processing and post-processing steps and their effects on prediction. The used dataset^42^ had been pre-processed in the following ways:

1. Functional pre-processing: Four different functional pre-processing pipelines were used: Connectome Computation System (CCS), Configurable Pipeline for the Analysis of Connectomes (CPAC), Data Processing Assistant for Resting-state fMRI (DPARSF), and Neuroimaging Analysis Kit (NIADK)
2. Processing Strategies: Each pre-processing was followed up by the usage (or not) of band-pass filtering and global signal correction. When applied a band-pass filtering of 0.01–0.1 Hz was used.
3. Regions of Interest (ROIs): We analyzed the time series extracted from 6 different ROI (i.e., Dosenbach 160, Craddock 200, Craddock 400, Eickhoff-Zilles, Harvard-Oxford, and Talaraich and Tournoux.)
4. Functional connectivity metric: We also explore four different ways to compute the functional connectivity interaction: covariance, correlation, tangent and partial correlation. These metrics were calculated using the nilearn package^43^.

The full space was composed of 384 pipelines (4 × 4 × 6 × 4 = 384; see Table S2 for an overview of the methods used).

### Constructing a low-dimensional space of pipelines

The steps to construct a low-dimensional space and search it are the same for the regression and classification problem, so they will only be described once.

In order to efficiently sample across a large number of pipelines, we need information about the general relationships between them. This is achieved by building a low-dimensional space that quantifies the similarity between pipelines in terms of a distance in the low-dimensional space (i.e., how similar is the obtained functional connectivity between the different approaches). Although the low-dimensional space should capture the similarity between approaches across a range of different problems and potentially a range of different datasets, we assessed the utility of building the space for two datasets using very different problem categories (i.e, regression and classification). However, the same space could be used to query different aspects of the dataset. For example, we used the regression space to ask questions about age, but it could equally be used to ask about other sources of individual variability (e.g., neuropsychiatric symptoms or cognitive ability).

**Fig. 8:**
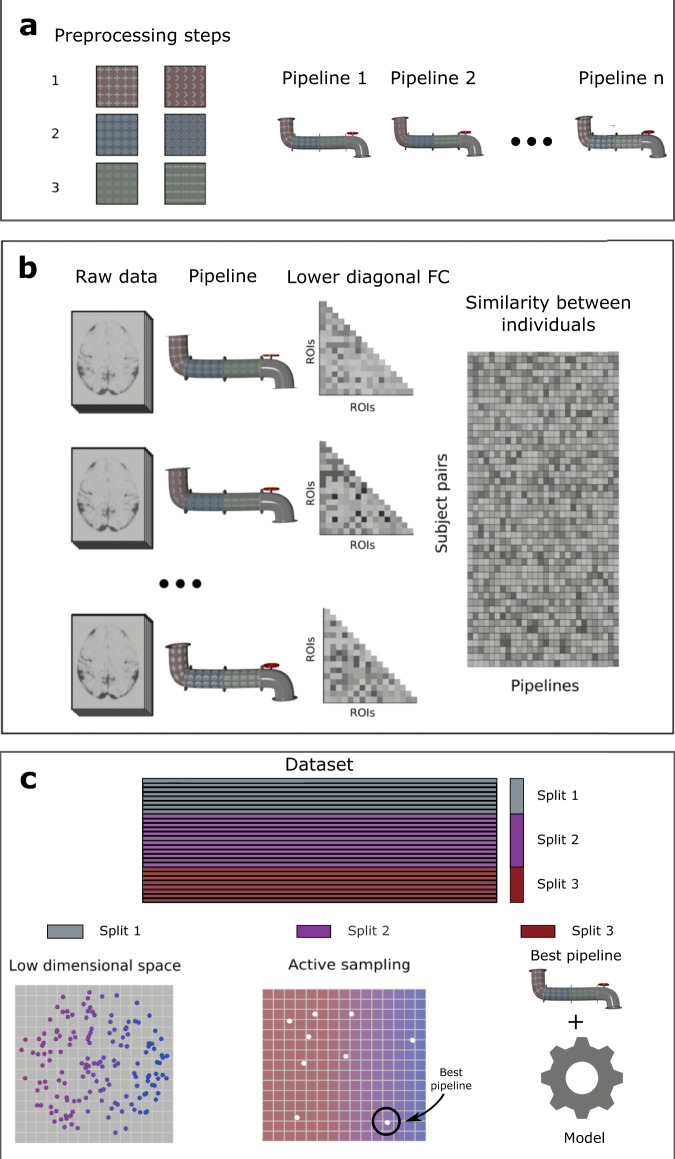
Overview of the methods to create a low-dimensional space of the multiverse of different pipelines. **a** The different analysis steps which encompassed different levels (ROI extraction, motion correction, functional connectivity metrics) were combined into one pipeline. The analysis steps are represented by the patterns and colors on the pipeline in the Figure. **b** The raw data was analyzed using different pipelines. For example, in the regression analysis the combination of global signal regression, 0.001 threshold, and betweenness centrality consist of one of the pipelines that was used process the raw data. The obtained data is then used to calculate the lower diagonal functional connectivity (FC) over all the regions of interest (ROIs) and used to create a region-to-region similarity. We then use the cosine similarity to identify the similarity between individual pairs and a specific pipeline and build the similarity matrix between individuals. **c** Schematic of how the different splits are used on the analysis. The datasets are divided into three splits. The first split is used to create the low dimensional space. Different embedding algorithms (e.g., MDS, t-SNE, PCA) are used to estimate the low-dimensional space from the flatted similarity between individuals. While split 2 was used during the active learning to identify the pipeline with the best performance, split 3 was used as a hold-out set to estimate the performance of the optimal pipeline identified with split 2.

To construct the low-dimensional space, we applied all pipelines (544 in the regression analysis and 384 for the classification analysis) to a subset of the participants’ individual FC data (50 randomly selected for the regression 176 individuals stratified by diagnosis for the classification analysis; Fig. 8). The aim was to build a space to locate approaches in terms of how they capture individual variability; therefore, for each approach, we calculated the cosine similarity matrix between pairs of participants. These were subsequently reshaped into a 2D matrix corresponding to between-participant distances (this led to a matrix of 1225 participant pairs by 544 pipelines for the regression analysis and a 15400 × 384 matrix for the classification. Because we ignore the self-similarity between subjects, the number of similarities can be computed by using n_subjects × (n_subjects − 1)/2).

Finally, the low-dimensional space was constructed with established embedding algorithms; we explored seven different algorithms: local-linear embedding^44^, spectral embedding^45^, t-distributed stochastic neighbor embedding (t-SNE)^46^, Uniform Manifold Approximation and Projection (UMAP)^47^, Potential of Heat-diffusion for Affinity-based Transition Embedding (PHATE)^48^, Principal Component Analysis (PCA), and multi-dimensional scaling (MDS)^49^. The objective of the embeddings was to create a space useful for active learning which would both: (i) capture similarity between approaches in terms of continuous distance in the space; as well as (ii) distribute approaches relatively evenly across the space. Based on observations of the spaces resulting from the aforementioned embedding algorithms, MDS was selected to use in subsequent active learning (see Results).

### Searching the space

Employing the low-dimensional space created using FC from a subsample of the data, active learning was used with the remaining participants, to sparsely sample the space in order to: (i) find the most successful approaches for predicting participant age and identify controls and autistic individuals based on FC; and, (ii) estimate age prediction or diagnosis ability for all models, including the large majority of models which were not sampled.

Active sampling is performed using closed-loop Bayesian optimization with Gaussian processes^50^. This loop involves: selecting a point in the space to sample; evaluating it in terms of 5-fold cross-validated predictive accuracy; fitting a Gaussian process (GP) regression to the space; and, evaluating an acquisition function using the GP regression to select the next point to sample. Although we use Bayesian optimization, the parameter *κ* determines the extent to which the sampling process is exploratory or exploitative; higher kappa values result in a much more detailed mapping of the space (reducing uncertainty across the whole space), versus finding best the point in the space.

When a point in the space is selected, the closest analysis approach to that point in the space is selected and its predictive accuracy evaluated by using support vector regression for the brain age prediction and a Logistic regression for the distinction between controls and autistic individuals, respectively. Gaussian process regression was implemented by the scikit-learn library^51,52^ using the RBF kernel and the default parameters. It is important to highlight that despite the prediction algorithm being constant, the input data varied depending on the selected analysis pipeline which could have, for example used a different method for motion correction, threshold or graph theory metric. We used split 2, to find the optimal pipeline (using negative mean absolute error and area under the receiver operating characteristic curve (AUC) for the regression and classification analysis, respectively) and evaluated the performance of the model and best pipeline obtained by evaluating it on split 3 (Fig. 8c). The metrics reported in Table 1 and Table 2 correspond to the model’s performance using the best pipeline identified by the active learning on the split 2 and evaluated on split 3 for 20 repetitions.

For the examples presented in the results, there was an initial burn-in phase in which ten points in the space were randomly selected and evaluated before active learning began. Bayesian optimization used the upper confidence bound (UCB) acquisition function^50^. The Gaussian process regression model used a Matern kernel combined with a white noise kernel, with kernel hyperparameters chosen in each iteration by maximizing log marginal likelihood using the default optimizer.

### Reporting summary

Further information on research design is available in the Nature Research Reporting Summary linked to this article.

## Supplementary information

Supplementary Information

Reporting Summary

## Data Availability

The data used for the regression analysis was previously released by Váša et al.^8^ and is available on Figshare (10.6084/m9.figshare.11551602). The pre-processed ABIDE dataset used in this study for the classification analysis can be downloaded from http://preprocessed-connectomes-project.org/abide/index.html. The data generated for creating the Figures shown in this study are provided in the Source Data file and can be obtained by running the provided code. Source data are provided with this paper.

## Source data

Source Data

## Code availability

The code used for analyses and figure generation is available on GitHub (https://github.com/Mind-the-Pineapple/into-the-multiverse) and can be run using Colab or jupyter notebooks.
