# Supplementary material for: A guided multiverse study of neuroimaging analyses
Source: Nat Commun. 2022 Jun 29;13:3758. doi: 10.1038/s41467-022-31347-8 (PMC9243029; doi:10.1038/s41467-022-31347-8)
Supplement: Supplementary file 1 — Supplementary Information [file 41467_2022_31347_MOESM1_ESM.pdf]

1 Supplementary Material

1.1 Regression Analysis

Table S1: **Regression Analysis:** Different analysis approaches used to create the pipelines to be evaluated. Every pipeline was composed of one type of regression data, one graph theory metric and a threshold leading to the creation of $2 \times 16 \times 17 = 544$ different analysis pipelines.

Data	Graph Theory Metric	Threshold
Motion regression	Degree	0.4
Global signal regression	Strength	0.3
	Betweenness-centrality	0.25
	Binary clustering coefficient	0.2
	Weighted clustering coefficient	0.175
	Eigenvector-centrality	0.15
	Subgraph-centrality	0.125
	Local efficiency	0.1
	Modularity (Louvain)	0.09
	Modularity (ProbTune)	0.08
	Participation coefficient	0.07
	Module Degree ZScore	0.06
	Pagerank-centrality	0.05
	Diversity coefficient	0.04
	Gateway degree	0.03
	K-core centrality	0.02
		0.01

1.1.1 Analysis of the Space Dissimilarity

To assess how much structure was preserved from the high-dimensional prediction space d to the lower-dimensional space $f_j(d) = \{y_i \mid y_i = f_j(x_i), y_i \in R^3\}$, we defined a measure of dissimilarity to quantify how the neighboring points are preserved after the manifold learning. The developed method relies on finding the k-Nearest Neighbors (kNN) in the high-dimensional space and evaluating if the kNN were maintained in the lower-dimensional space. A high dissimilarity score means that the nearest models were not maintained in the new manifold. Because the neighboring points might change depending on the number of neighbors analyzed, we iterated over different values which represented the k-number of neighbors in order to assess which manifold learning algorithm best maintained the topological information present in the high-dimensional space. Due to computational limitations we could not calculate the dissimilarity of the entire space, therefore, we sampled 100 models over 100 repetitions for $k \in \{2, .., N/2\}$[1] to guarantee that all models were sampled at least once for every k. We define the set of indices that corresponds to all nearest neighbors as

$$A(d) = \{(i, j) \mid x_i \in kNN(x_j) \ and \ x_j, x_i \in d\} \ , \tag{1}$$

where kNN(x_j) is the set of k Nearest Neighbors of x_j with respect to the L^2 norm. We then apply above definition on $f_j(d)$ and specify $A(f_j(d))$.

We defined the dissimilarity ϵ_k as

$$\epsilon_k = \frac{\mid A(f_j(d)) \setminus A(d) \mid}{k \ n} \ , \tag{2}$$

where $| \cdot |$ is the cardinality (i.e., the number of elements in a set), n the number of sampled points from d, and $\setminus$ the difference operators in set theory (i.e., A $\setminus$ B consists of a set composed by all elements that are in

[1]We restrict k to $N/2$ due to combinatorial reasons.

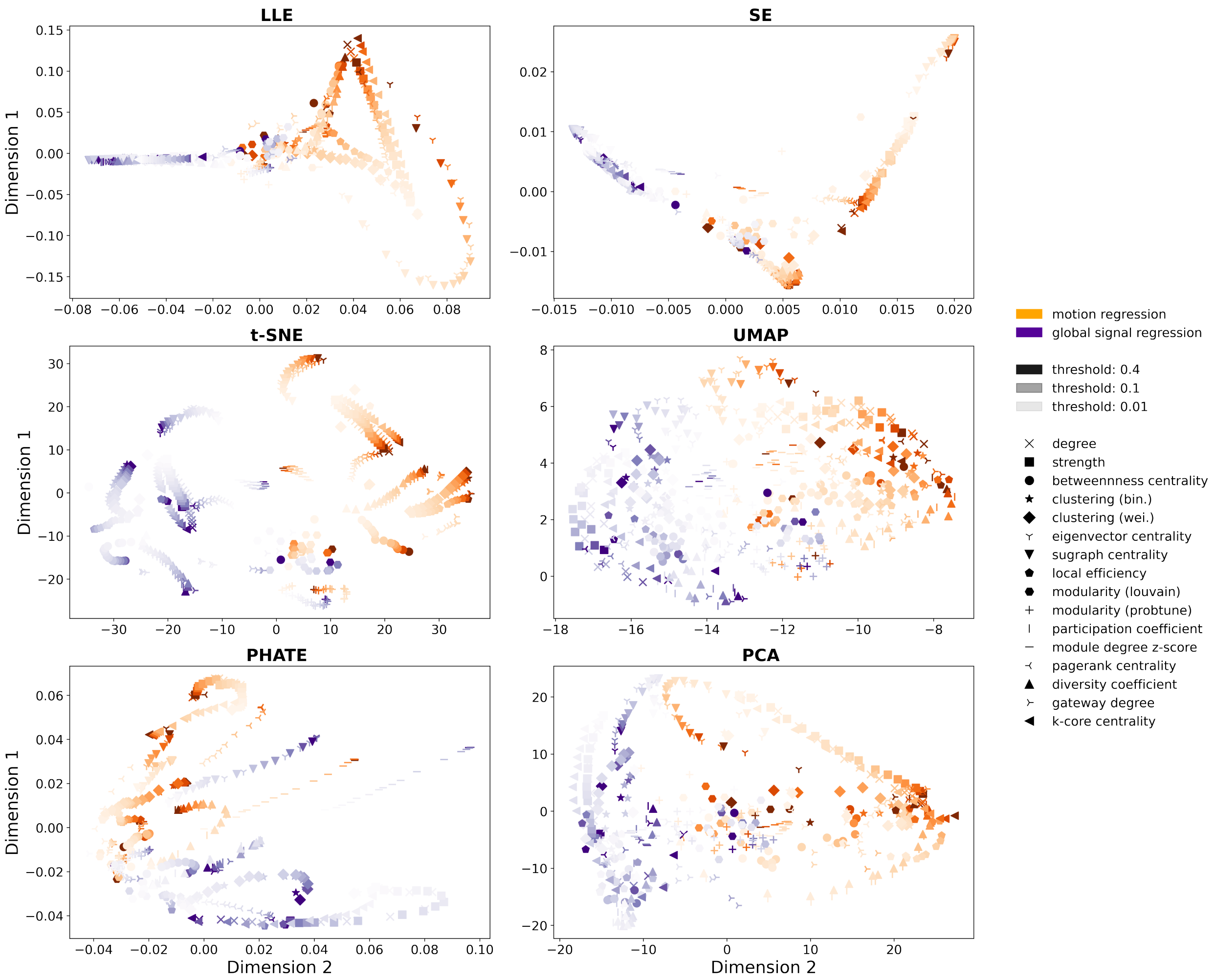

Figure S1: **Regression Analysis:**The low-dimensional space for different embedding approaches using the regression dataset. Similar to Figure 1 in the main manuscript, each point correspond to a pipeline that was built using different regressions, graph theory thresholds and metrics. The figure illustrates the built space for the following approaches: Local linear embedding (LLE), Spectral embedding (SE), t-Stochastic Neighbor Embedding (t-SNE), Uniform Manifold Approximation, Projection (UMAP), Potential of Heat-diffusion for Affinity-based Transition Embedding (PHATE) and Principal Component Analysis (PCA).

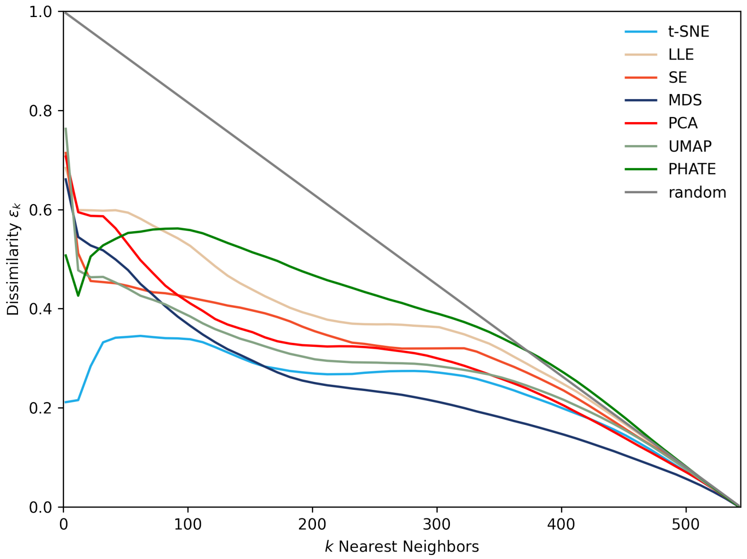

Figure S2: **Regression Analysis:**Dissimilarity score of the different low-dimensional embedding. The dissimilarity value quantifies how much the neighborhoods were maintained from the original to the reduced space; this was used to assess how much the different embedding algorithms maintained the topological information present in the original space. The dissimilarity score can assume values between 0 and 1; while small values mean that the neighborhood was similar after the embedding, values close to one signify that the neighborhood was not conserved after the embedding. LLE, SE and PCA showed high dissimilarity values compared to MDS. Although t-SNE and UMAP showed a good similarity for a small number of neighbors, the MDS algorithm was able to keep the closest similarity for larger neighborhoods. The gray line represents the dissimilarity score that would be achieved if all the neighbors would be randomly shuffled. This nicely illustrates that for very large number of clusters, the similarity scores are very close to the random performance.

set A but not B). This expression counts the indices that were present in the original space but not in the lower-dimensional representation and is normalized by the total numbers of kNN in the original space and n. The dissimilarity metric can assume values between 0 and 1; where 0 means that the kNN were maintained after the manifold learning and 1 means that all the kNN were different.

Because MDS had the lowest dissimilarity with the original space, we used the lower dimensional space created by this approach in further analysis.

1.2 Classification Analysis

Table S2: **Classification Analysis:** Different analysis approaches used to create the pipelines to be evaluated for the classification analysis. Every pipeline was composed of one type of functional data pre-processing, one processing strategy, one set of regions of interest and one connectivity metric leading to the creation of 384 different analysis pipelines. Abbreviations: CCS (Connectome Computation System); CPAC (Configurable Pipeline for the Analysis of Connectomes); DPARSF (Data Processing Assistant for Resting-State fMRI); NIAK (Neuroimaging Analysis Kit); filt (Band-pass filtering (0.01 - 01 Hz); global and noglobal (Global signal regression and no global signal regression, respectively)).

Functional Pre-processing	Processing Strategy	Regions of Interest	Connectivity Metric
CSS	filt_global	Eickhoff-Zilles (ez)	Correlation
CPAC	filt_noglobal	Harvard-Oxford (ho)	Covariance
DPARSF	nofilt_global	Talaraich and Tournoux (tt)	Partial Correlation
NIAK	nofilt_noglobal	Dosenbach 160	Tangent
		Craddock 200 (cc200)	
		Craddock 400 (cc400)	

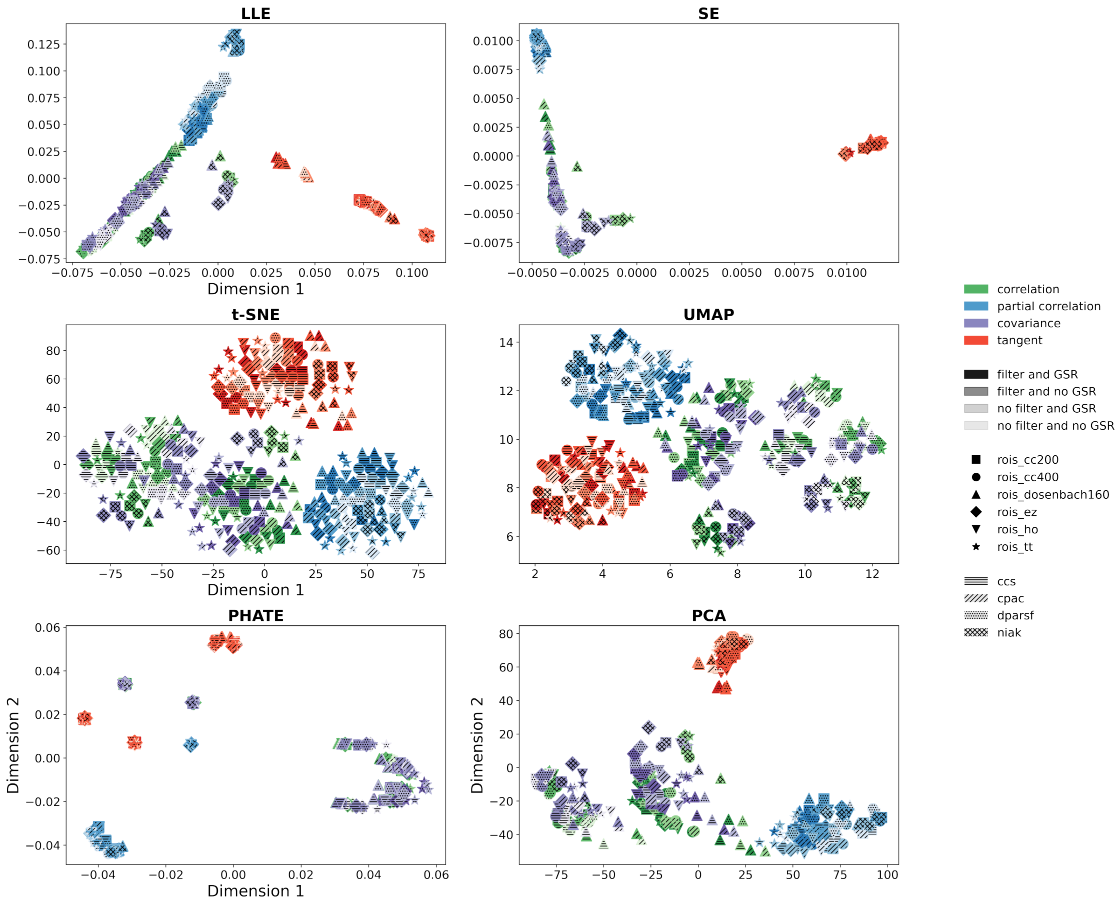

Figure S3: **Classification Analysis:** Low-dimensional embeddings obtained using four types of embedding: Local linear embedding (LLE), Spectral embedding (SE), t-Stochastic Neighbor Embedding (t-SNE) and Uniform Manifold Approximation and Projection (UMAP), Potential of Heat-diffusion for Affinity-based Transition Embedding (PHATE) and Principal Component Analysis (PCA).
