# Supplementary material for: A guided multiverse study of neuroimaging analyses
Source: Nat Commun. 2022 Jun 29;13:3758. doi: 10.1038/s41467-022-31347-8 (PMC9243029; doi:10.1038/s41467-022-31347-8)
Supplement: Supplementary file 2 — Reporting Summary [file 41467_2022_31347_MOESM2_ESM.pdf]

nature research

Corresponding author(s): Jessica Dafflon and Robert Leech

Last updated by author(s): Feb 12, 2022

Reporting Summary

Nature Research wishes to improve the reproducibility of the work that we publish. This form provides structure for consistency and transparency in reporting. For further information on Nature Research policies, see our Editorial Policies and the Editorial Policy Checklist.

Statistics

For all statistical analyses, confirm that the following items are present in the figure legend, table legend, main text, or Methods section.

n/a	Confirmed	
☐	☒	The exact sample size (n) for each experimental group/condition, given as a discrete number and unit of measurement
☐	☒	A statement on whether measurements were taken from distinct samples or whether the same sample was measured repeatedly
☒	☐	The statistical test(s) used AND whether they are one- or two-sided<br>*Only common tests should be described solely by name; describe more complex techniques in the Methods section.*
☒	☐	A description of all covariates tested
☒	☐	A description of any assumptions or corrections, such as tests of normality and adjustment for multiple comparisons
☐	☒	A full description of the statistical parameters including central tendency (e.g. means) or other basic estimates (e.g. regression coefficient) AND variation (e.g. standard deviation) or associated estimates of uncertainty (e.g. confidence intervals)
☒	☐	For null hypothesis testing, the test statistic (e.g. F, t, r) with confidence intervals, effect sizes, degrees of freedom and P value noted<br>*Give P values as exact values whenever suitable.*
☒	☐	For Bayesian analysis, information on the choice of priors and Markov chain Monte Carlo settings
☐	☒	For hierarchical and complex designs, identification of the appropriate level for tests and full reporting of outcomes
☐	☒	Estimates of effect sizes (e.g. Cohen's d, Pearson's r), indicating how they were calculated

Our web collection on statistics for biologists contains articles on many of the points above.

Software and code

Policy information about availability of computer code

Data collection

The data was not acquired during this study, therefore no data collection software was used.

Data analysis

All the analysis were conducted with Python. The code is available on GitHub (https://github.com/Mind-the-Pineapple/into-the-multiverse) and can be run by anyone using the provided Colab notebook and jupyter notebook. By running the Colab notebook shared on the studies GitHub all figures can be regenerated.

The following versions of the software were used:
Python 3.7.12

scikit-learn==0.24.1
pyreadr==0.2.9
scipy==1.5.2
bctpy==0.5.2
matplotlib==3.3.0
numpy==1.19.1
bayesian-optimization==1.2.0
umap-learn==0.4.6
phate==1.0.7
nilearn==0.7.0
nibabel==3.2.1

For manuscripts utilizing custom algorithms or software that are central to the research but not yet described in published literature, software must be made available to editors and reviewers. We strongly encourage code deposition in a community repository (e.g. GitHub). See the Nature Research guidelines for submitting code & software for further information.

Data

Policy information about availability of data

All manuscripts must include a data availability statement. This statement should provide the following information, where applicable:

- Accession codes, unique identifiers, or web links for publicly available datasets
- A list of figures that have associated raw data
- A description of any restrictions on data availability

The regression analysis data was previously released by Váša et al. (https://www.pnas.org/content/117/6/3248) and is available on Figshare (https://doi.org/10.6084/m9.figshare.11551602) and the data for classification is available at (http://preprocessed-connectomes-project.org/abide/download.html) .

Field-specific reporting

Please select the one below that is the best fit for your research. If you are not sure, read the appropriate sections before making your selection.

☒ Life sciences ☐ Behavioural & social sciences ☐ Ecological, evolutionary & environmental sciences

For a reference copy of the document with all sections, see nature.com/documents/nr-reporting-summary-flat.pdf

Life sciences study design

All studies must disclose on these points even when the disclosure is negative.

Sample size	We used all subject data provided by Ýša et al and the ABIDE consortia, no exclusions. No power calculation was needed in advance and we used all samples available.
Data exclusions	Quality tests have been conducted in the previous studies, as reported in Váša et al. (https://www.pnas.org/content/117/6/3248) and the Preprocessed Connectome Project (http://preprocessed-connectomes-project.org/quality-assessment-protocol/). We made no further changes or exclusions to the data.
Replication	As this is a machine learning study where the code is provided, the entire analysis can be replicated at anytime by anyone. Anyone interested in replicating can use the provided Colab (https://colab.research.google.com/github/Mind-the-Pineapple/into-the-multiverse/blob/master/notebooks/multiverse_analysis.ipynb) or using jupyter notebook to run the analysis. Furthermore, we replicated the approach using two independent datasets.
Randomization	Regression analysis:<br>The data was randomly split. While 50 individuals were used to build the low-dimensional space, the 198 individuals were used to perform the search and the remaining 50 individuals were as a holdout dataset.<br>Classification analysis:<br>The data was randomly split. While 176 individuals were used to build the low-dimensional space, the 529 subjects were used to create the space and the remaining 177 were used to validate the performance on a hold-out dataset.
Blinding	This analyses do not require blinding as there were no interventions or group analysis. However, the framework includes a step to randomly split the data into train and test sets, and this process was repeated multiple times to check the splits did not affects the results.

Reporting for specific materials, systems and methods

We require information from authors about some types of materials, experimental systems and methods used in many studies. Here, indicate whether each material, system or method listed is relevant to your study. If you are not sure if a list item applies to your research, read the appropriate section before selecting a response.

Materials & experimental systems

n/a	Involved in the study	
☒	☐	Antibodies
☒	☐	Eukaryotic cell lines
☒	☐	Palaeontology and archaeology
☒	☐	Animals and other organisms
☐	☒	Human research participants
☒	☐	Clinical data
☒	☐	Dual use research of concern

Methods

n/a	Involved in the study	
☒	☐	ChIP-seq
☒	☐	Flow cytometry
☐	☒	MRI-based neuroimaging

Human research participants

Policy information about studies involving human research participants

Population characteristics	See above
Recruitment	No data was acquired during the study for a detailed description of the recruitment for the regression analysis see (https://www.pnas.org/content/117/6/3248) and for the classification analysis see (https://www.nature.com/articles/mp201378#Sec2)
Ethics oversight	Regression analysis:<br>Details on the ethical approval and oversight for the data acquisition can be found in the Váša et al. paper (https://www.pnas.org/content/117/6/3248)<br>Classification analysis:<br>Details on the ethical approval and oversight for the data acquisition can be found in the Di Martino et al. paper (https://www.nature.com/articles/mp201378#Sec2)

Note that full information on the approval of the study protocol must also be provided in the manuscript.

Magnetic resonance imaging

Experimental design

Design type	resting state fMRI
Design specifications	Regression analysis:<br>The data used consisted of one scan per participant each scan lasted 10min.<br>Classification analysis:<br>The design specifications can be found at https://www.nature.com/articles/mp201378
Behavioral performance measures	No behavior data was acquired during the scan.

Acquisition

Imaging type(s)	functional
Field strength	3T
Sequence & imaging parameters	Regression analysis:<br>The sequence and imaging parameters are fully described at https://www.pnas.org/content/pnas/suppl/2020/01/27/1906144117.DCSupplemental/pnas.1906144117.sapp.pdf.<br>Classification analysis:<br>The sequence and imaging parameters are fully described at http://fcon_1000.projects.nitrc.org/indi/abide/abide_I.html
Area of acquisition	Regression analysis:<br>The following was taken form the original manuscript that describes the data (https://www.pnas.org/content/pnas/suppl/2020/01/27/1906144117.DCSupplemental/pnas.1906144117.sapp.pdf):<br>Each participant's cortex was parcellated using two parcellations: a recent multi-modal parcellation into 360 bilaterally symmetric regions based on data from the Human Connectome Project, as well as a sub-parcellation of the Desikan-Kiliany anatomical atlas into 308 parcels of approximately equal surface area (?5cm2). Additionally, subcortical regions were provided by FreeSurfer software, and included bilateral pairs of the following regions: thalamus, caudate, putamen, pallidum, hippocampus, amygdala, nucleus accumbens and the ventral diencephalon. Thus, the combination of each of the two cortical parcellations with the subcortical regions yielded parcellations of cortex and subcortex into 376 regions (henceforth referred to as the HCP parcellation) and 324 regions (henceforth referred to as the DK-sub parcellation) respectively.<br>Classification analysis:<br>Different parcellations were used and compared on the manuscript. For a general overview of the different parcellation methods used please refer to http://preprocessed-connectomes-project.org/abide/Pipelines.html

Diffusion MRI ☐ Used ☒ Not used

Preprocessing

Preprocessing software	As this is part of the question being investigated in the manuscript, different additional preprocessing steps (i.e., dimensionality reduction) are described in the manuscript.<br>Regression analysis:<br>The data we received was preprocessed using AFNI and Freesurfer (v5.3.0).<br>Classification analysis:<br>The data has been pre-processed using different frameworks. For an overview of all frameworks used please refer to http://preprocessed-connectomes-project.org/abide/Pipelines.html

Normalization	Regression<br>We used the preprocessed an normalized data provided by Váša et al., which was normalized using the default pipeline provided by Freesurfer (v5.3.0).<br>Classification:<br>The used dataset had been normalised by the Preprocessed Connectomes Project (PCP). Information on how the normalization was performed can be found at http://preprocessed-connectomes-project.org/abide/
Normalization template	Regression analysis:<br>The standard average template from Freesurfer was used followed by a parcellating the data into the Desikan-Kiliany anatomical atlas augmented by subcortical regions defined by Freesurfer.<br>Classification<br>All the data was transformed into the MNI152 template
Noise and artifact removal	Regression analysis:<br>This is fully described on from Váša et al supplementary materials (https://www.pnas.org/content/pnas/suppl/2020/01/27/1906144117.DCSupplemental/pnas.1906144117.sapp.pdf)<br>Classification:<br>Information can be found at http://fcon_1000.projects.nitrc.org/indi/abide/abide_I.html
Volume censoring	Regression analysis:<br>This is fully described on from Váša et al supplementary materials (https://www.pnas.org/content/pnas/suppl/2020/01/27/1906144117.DCSupplemental/pnas.1906144117.sapp.pdf)<br>Classification:<br>Information can be found at http://fcon_1000.projects.nitrc.org/indi/abide/abide_I.html and http://preprocessed-connectomes-project.org/quality-assessment-protocol/

Statistical modeling & inference

Model type and settings	The paper focus on a new analysis method by creating a low dimensional space and using active inference to navigate the created space.
Effect(s) tested	Regression:<br>We are using whole brain to predict brain age.<br>Classification:<br>We use functional connectivity to classify between controls and individuals with autism diagnosis.<br><br>However, the focus of the paper is not on the age prediction or the classification between controls and individuals with autism diagnosis, but on the development of a new method and we use datasets as illustrative cases.

Specify type of analysis: ☐ Whole brain ☒ ROI-based ☐ Both

Anatomical location(s)	Regression:<br>The standard average template from Freesurfer was used followed by a parcellating the data into the Desikan-Kiliany anatomical atlas augmented by subcortical regions defined by Freesurfer.<br>Classification:<br>We used six different methods to extract functional connectivity. A list of the methods is provided in the manuscript on Table S2.

Statistic type for inference<br>(See Eklund et al. 2016)	Statistics derived from graph theoretic measures to predict brain age (regression analysis) and functional connectivity obtained from different methods to classify between controls and individuals with autism spectrum disorder.
Correction	We used a hold-out dataset for the prediction analysis

Models & analysis

n/a	Involved in the study
☐	☒ Functional and/or effective connectivity
☐	☒ Graph analysis
☐	☒ Multivariate modeling or predictive analysis

Functional and/or effective connectivity	Functional connectivity calculated using Person's correlation, partial correlation, tangent and covariance.
Graph analysis	The choice of graph analysis methods is one of the focus of the paper and is described in detail in the paper.
Multivariate modeling and predictive analysis	The choice of multivariate modeling is one of the focus of the paper and is described in detail in the paper.
